# Bacterial dynamin homolog regulates growth phase-dependent c-di-GMP production

**DOI:** 10.1128/mbio.00668-26

**Published:** 2026-07-20

**Authors:** Xuanlin Chen, Ronja Offer, Verena Maria Suchanek, Regine Hengge, Victor Sourjik

**Affiliations:** 1 Department of Systems and Synthetic Biology, Max Planck Institute for Terrestrial Microbiology and Center for Synthetic Microbiology (SYNMIKRO) 28310, Marburg, Germany; 2 Institut für Biologie/Mikrobiologie, Humboldt-Universität zu Berlin 9373, Berlin, Germany; University of Maryland, College Park, Maryland, USA

**Keywords:** bacteria, c-di-GMP, second messenger, biofilms, dynamin, energy state, GTP, ppGpp

## Abstract

The bacterial second messenger bis-(3′-5′)-cyclic dimeric guanosine monophosphate (c-di-GMP) governs the transition from motile to sessile lifestyle by suppressing flagellar motility and promoting the synthesis of the extracellular biofilm matrix. In *Escherichia coli*, a main contributor to the global c-di-GMP pool is the diguanylate cyclase DgcE. It was recently shown that DgcE is activated by physical interaction with the dynamin-like GTPase RdcA and its accessory protein RdcB, but molecular details and physiological relevance of this regulation have not been fully revealed. Here, we used a combination of *in vitro* and *in vivo* experiments to characterize the complex formation between RdcA, RdcB, and DgcE. We corroborate that RdcA functions as a modular GTP sensor to control the activity of DgcE; while its C-terminal domain binds to and activates DgcE, the N-terminal GTPase domain confers regulation. We show that a drop in GTP levels triggers RdcA localization to the cytoplasmic membrane and its interaction with DgcE. RdcB coordinates the formation of larger RdcA-DgcE assemblies, and it is required for the DgcE activation by RdcA. Additionally, we demonstrate that the alarmone (p)ppGpp modulates the DgcE activation by counteracting the inhibitory effect of GTP on RdcA activity. Taken together, our findings further characterize a sophisticated regulatory mechanism of c-di-GMP signaling in *E. coli*, in which the ternary RdcA-RdcB-DgcE complex integrates GTP and (p)ppGpp signals to monitor amino acid starvation and/or cellular energy status, thus controlling transition to a multi-cellular, biofilm-associated lifestyle.

**IMPORTANCE:**

As a central regulator of motile-to-sessile lifestyle switching, bis-(3′-5′)-cyclic dimeric guanosine monophosphate (c-di-GMP) needs to be dynamically modulated in response to growth phase transitions and environmental challenges. Our work characterizes a novel post-translational mechanism of c-di-GMP regulation, where a bacterial GTPase, RdcA, that is homologous to eukaryotic dynamins, couples the physiological state of cells to c-di-GMP production. This coupling includes a cell energy- or nutrient-dependent recruitment of RdcA to the cytoplasmic membrane, where it interacts with and activates a major c-di-GMP producing enzyme. In contrast to the established function of eukaryotic and bacterial dynamins in membrane remodeling, our study establishes an example of a dynamin-like protein functioning as a GTP-sensitive switch and trigger enzyme that controls second messenger signaling in bacteria.

## INTRODUCTION

Nucleotide-based second messengers mediate cell signaling across all domains of life. In bacteria, bis-(3′-5′)-cyclic dimeric guanosine monophosphate (c-di-GMP) acts as a central regulator of the transition from a motile planktonic lifestyle to the sessile biofilm lifestyle (1–3). High c-di-GMP levels inhibit flagellar motility and activate biofilm matrix production by binding to a multitude of effectors, including regulatory proteins, enzymes, and riboswitches (4, 5). To enable lifestyle switching in response to various environmental changes, c-di-GMP levels in most bacteria are controlled by a large number of antagonistic diguanylate cyclases (DGCs) and c-di-GMP-specific phosphodiesterases (PDEs), which synthesize and degrade c-di-GMP, respectively (6). While these enzymes share conserved catalytic domains—the GGDEF motif in DGCs and the EAL or HD-GYP domain in PDEs—their N-terminal sensor domains are highly variable, which enables the regulation of intracellular c-di-GMP levels in response to different stimuli (6–8).

In *Escherichia coli* K-12, this network consists of 12 DGCs and 13 c-di-GMP-specific PDEs (9). The major pathway controlled by c-di-GMP in *E. coli* is the synthesis of the extracellular biofilm matrix components, including proteinaceous curli fibers (10), pEtN-cellulose (11, 12), and poly-β-1,6-*N*-acetylglucosamine (PGA) (13). Biofilm matrix production is inversely regulated with flagellar activity and is activated via a complex signaling cascade centered around the master regulator of the curli operon, CsgD, when cells enter the stationary phase (14). Upon this entry, the activity of the flagellar-specific alternative sigma factor FliA is reduced, which leads to reduced expression of PdeH, the FliA-dependent master PDE of *E. coli*, alleviating c-di-GMP degradation and thus leading to its increased levels (14). Simultaneously, the increased level of activity of the stationary-phase sigma factor RpoS induces the expression of several DGCs, ultimately resulting in elevated c-di-GMP levels (9).

The top-level positive regulator in this c-di-GMP signaling cascade is DgcE, which is the major and, structurally, the most complex DGC in *E. coli* K-12. It consists of a membrane-embedded MASE1 domain, followed by three PAS/PAC domains, an active GGDEF domain, and a degenerate EAL domain. The MASE1 and the PAS/PAC domains are essential for its enzymatic activity by promoting dimerization, whereas the EAL domain exerts a negative regulation, possibly by preventing the dimerization of the GGDEF domains (15), which is essential for the condensation of two GTP molecules into c-di-GMP (6). This modular architecture indicated that DgcE activity might be responsive to complex, yet poorly understood, post-translational regulation.

**Fig 1:**
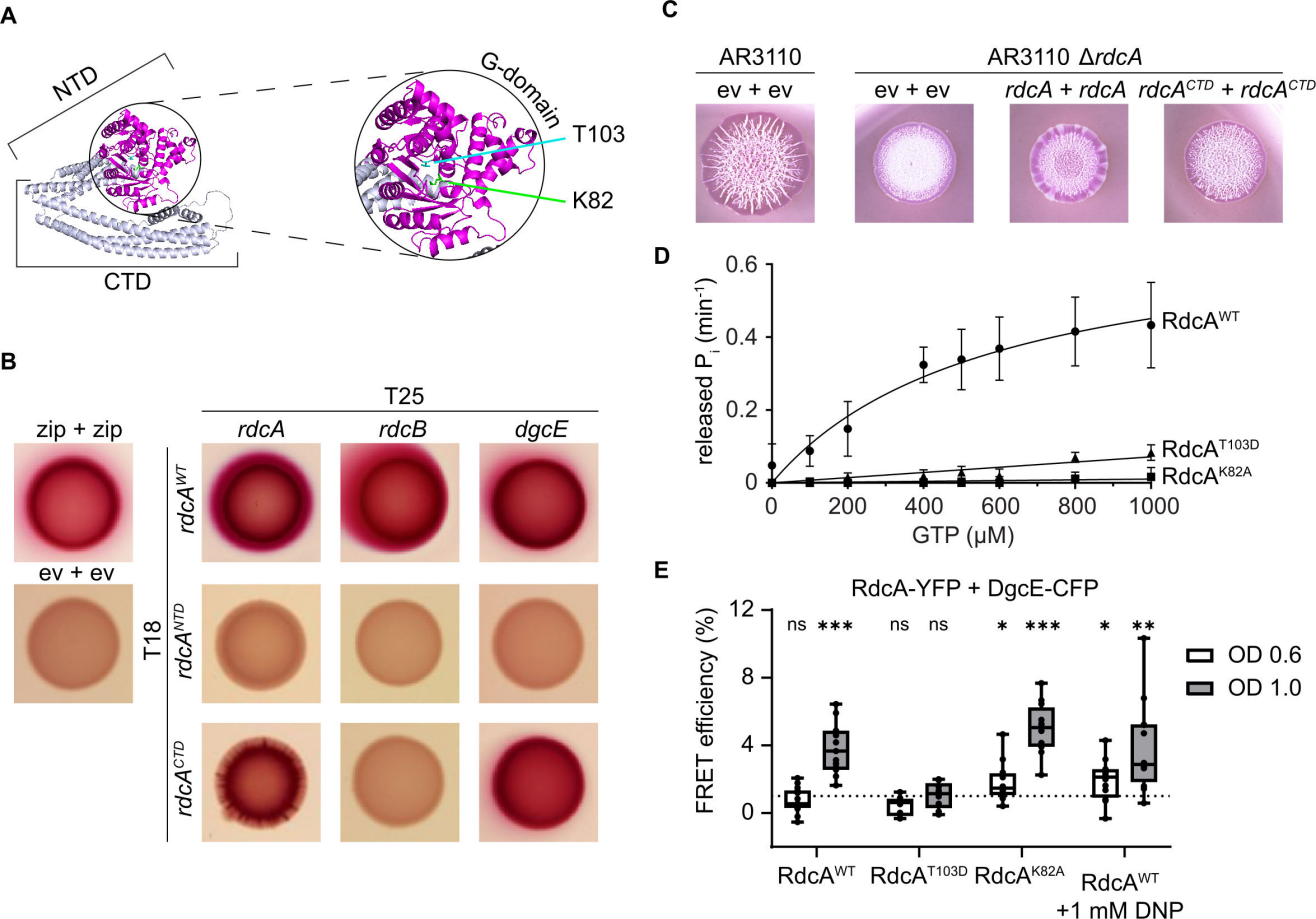
Determinants of RdcA interaction with DgcE. (**A**) Predicted structure of RdcA generated by AlphaFold3, depicting the C-terminal and N-terminal domains (CTD and NTD, respectively). The GTPase sub-domain within the NTD is highlighted in magenta. Key residues for GTP hydrolysis (T103, cyan) and GTP binding (K82, green) are indicated. (**B**) Analysis of RdcA domain interactions with DgcE and RdcB using an adenylate cyclase-based bacterial two-hybrid (BACTH) assay, with red color indicating interaction. The leucine zipper protein (zip) dimer and empty vectors (ev) were used as positive and negative controls, respectively. (**C**) Macrocolony morphology of the AR3110 wild-type strain and the ∆*rdcA* mutant complemented with plasmid-encoded full-length *rdcA* or *rdcA*^CTD^ on Congo red plates for 5 days at 28°C. Δ*rdcA* was complemented with two plasmids expressing *rdcA* to make the experiment equivalent to the BACTH assay. (**D**) GTPase activity of purified RdcA and indicated mutant proteins. Release of inorganic phosphate (Pi) was measured using the malachite green assay after a 1-h incubation of purified protein with indicated GTP concentrations at 37°C. Data are normalized to protein amount and represent the mean ± SD (*n* = 3). (**E**) Interaction between DgcE-CFP and RdcA-YFP or indicated mutants quantified using acceptor photobleaching-based Förster resonance energy transfer (FRET) assay. DgcE-CFP and RdcA-YFP were expressed from pBAD33 and pTrc99a plasmids, respectively, induced with 0.02% L-arabinose and 30 µM IPTG. Relative extent of complex formation between two proteins is reflected by FRET efficiency. Data are presented as boxplots with individual data points, with each point representing a technical replicate from 3 to 5 biological replicates. Statistical significance was determined by a one-tailed *t*-test against 1% (dashed line), a previously established threshold for specific protein interactions (see Materials and Methods) (*P < 0.05, **P < 0.005, ***P < 0.0005; “ns,” not significant).

A previous study (15) has established that one such regulatory input to stimulate DgcE activity is provided by the bacterial dynamin-like protein RdcA. Together with its accessory protein RdcB, RdcA activates DgcE in a manner that is controlled by GTP binding and hydrolysis. The dynamin superfamily of GTPases is well known to be involved in membrane remodeling in eukaryotes (16), whereas their functions in bacteria appear to be diverse and were only established in a few cases (17–20). RdcA possesses a canonical N-terminal GTPase domain, with all conserved motifs for GTP binding and hydrolysis (21) (Fig. 1A). However, unlike classical dynamins or the bacterial dynamin-like protein in *Nostoc punctiforme*, which respectively localize to the membrane via a pleckstrin homology domain or a hydrophobic paddle (16, 20), RdcA lacks any known membrane-binding domain (21).

In this work, we further characterized the mechanism and the physiological significance of DgcE regulation by RdcA. Our results corroborate that RdcA functions as a sensor of GTP levels, with the binding of GTP inhibiting recruitment of RdcA to the cytoplasmic membrane and, consequently, its binding to the membrane-located DgcE. The activation of DgcE by RdcA additionally requires RdcB and might involve the formation of supramolecular complexes between these three proteins. Furthermore, we demonstrate modulation of RdcA activity by competitive binding of the alarmone (p)ppGpp. Together, such RdcA-dependent activation of DgcE by low GTP and high (p)ppGpp levels is essential for c-di-GMP upregulation in *E. coli* during transition to the stationary phase.

## RESULTS

### RdcA interaction with DgcE is mediated by the C-terminal domain and regulated by the N-terminal domain

To identify functional determinants of RdcA implicated in the formation of the complex between RdcA, RdcB, and DgcE, we first used a bacterial two-hybrid (BACTH) assay. BACTH is based on the functional reconstitution of the split adenylate cyclase (CyaA), which enables an *E. coli* Δ*cyaA* strain to ferment lactose and results in red colonies on MacConkey agar plates (22, 23). Consistent with the previous findings (15), full-length RdcA (RdcA^WT^) exhibited strong interactions with RdcB, DgcE, and itself in this assay (Fig. 1B). By testing the truncated versions of RdcA, we showed that the N-terminal domain (RdcA^NTD^) failed to interact with any of these partners, whereas the C-terminal domain (RdcA^CTD^) retained the ability to homodimerize and interact with DgcE. However, neither fragment alone interacted with RdcB. These results imply that RdcA^CTD^ is the primary domain responsible for engaging DgcE, whereas the interaction with RdcB apparently requires both domains.

We next sought to assess whether the physical interaction between RdcA^CTD^ and DgcE is sufficient to stimulate the DgcE activity and therefore biofilm matrix production. Here, we used a macrocolony assay on Congo red plates where the production of curli fibers and pEtN-cellulose leads to the binding of Congo red dye and colony wrinkling (11). Consistent with reduced c-di-GMP levels in the absence of DgcE activation, deletion of *rdcA* resulted in smooth, white colonies indicating defective matrix production (Fig. 1C). The expression of RdcA^CTD^ alone partially rescued matrix formation, although it was less efficient than complementation by the full-length RdcA. Thus, RdcA^CTD^ is sufficient not only to interact with but also to at least partly activate DgcE.

To better understand the functional role of the GTPase RdcA^NTD^, we first assessed the enzymatic activity of purified RdcA. The wild-type RdcA hydrolyzed GTP to GDP and Pi, as detected using the malachite green assay, with a *K*_*m*_ of 579 µM and a *V*_*max*_ of 0.7 min^−1^ (Fig. 1D). Such low affinity and low GTPase activity suggest that RdcA may operate as a sensory switch in the physiological range of the GTP concentrations, as these decline during *E. coli* transition to the stationary phase (24, 25). In addition to the wild-type protein, we tested RdcA variants with amino acid substitutions at residues that are conserved in dynamin GTPases: lysine residue (K82) required for GTP binding and threonine residue (T103) required for GTP hydrolysis (15) (Fig. 1A). As expected, the GTPase activity was abolished in both protein variants.

We next investigated whether the interaction between RdcA and DgcE is growth phase-dependent using FRET, which can monitor complex formation between fluorescently tagged proteins. Functionality of DgcE fused to cyan fluorescent protein (DgcE-CFP) and RdcA fused to yellow fluorescent protein (RdcA-YFP) has been validated previously (26). While we observed significant interaction between DgcE-CFP and RdcA-YFP in the early stationary-phase cells grown to an optical density (OD) of 1.0, the FRET signal in cells harvested at the exponential phase (OD 0.6) remained below the previously established significance threshold for specific binding, 0.5%–1% (27, 28) (Fig. 1E; Fig. S1A and B). The hydrolysis-deficient, and thus constitutively GTP-bound, variant (RdcA^T103D^) failed to show a significant FRET signal at either growth phase, confirming the inhibitory role of GTP binding on the interaction between RdcA and DgcE. In contrast, the GTP-free variant (RdcA^K82A^) showed significant interaction with DgcE even during exponential growth, although this interaction was further enhanced in later stages of growth. Deenergizing cells using the uncoupling agent 2,4-dinitrophenol (DNP), which should deplete cellular GTP levels, was sufficient to induce RdcA-DgcE interaction even at exponential phase (OD 0.6). This further supports our hypothesis that the growth-phase dependence of the RdcA-DgcE interaction could be at least partly explained by the variation in cellular GTP levels. Nevertheless, other factors may additionally contribute to the enhancement of this interaction in early stationary-phase cells.

### Cellular GTP levels control the membrane association of RdcA

**Fig 2:**
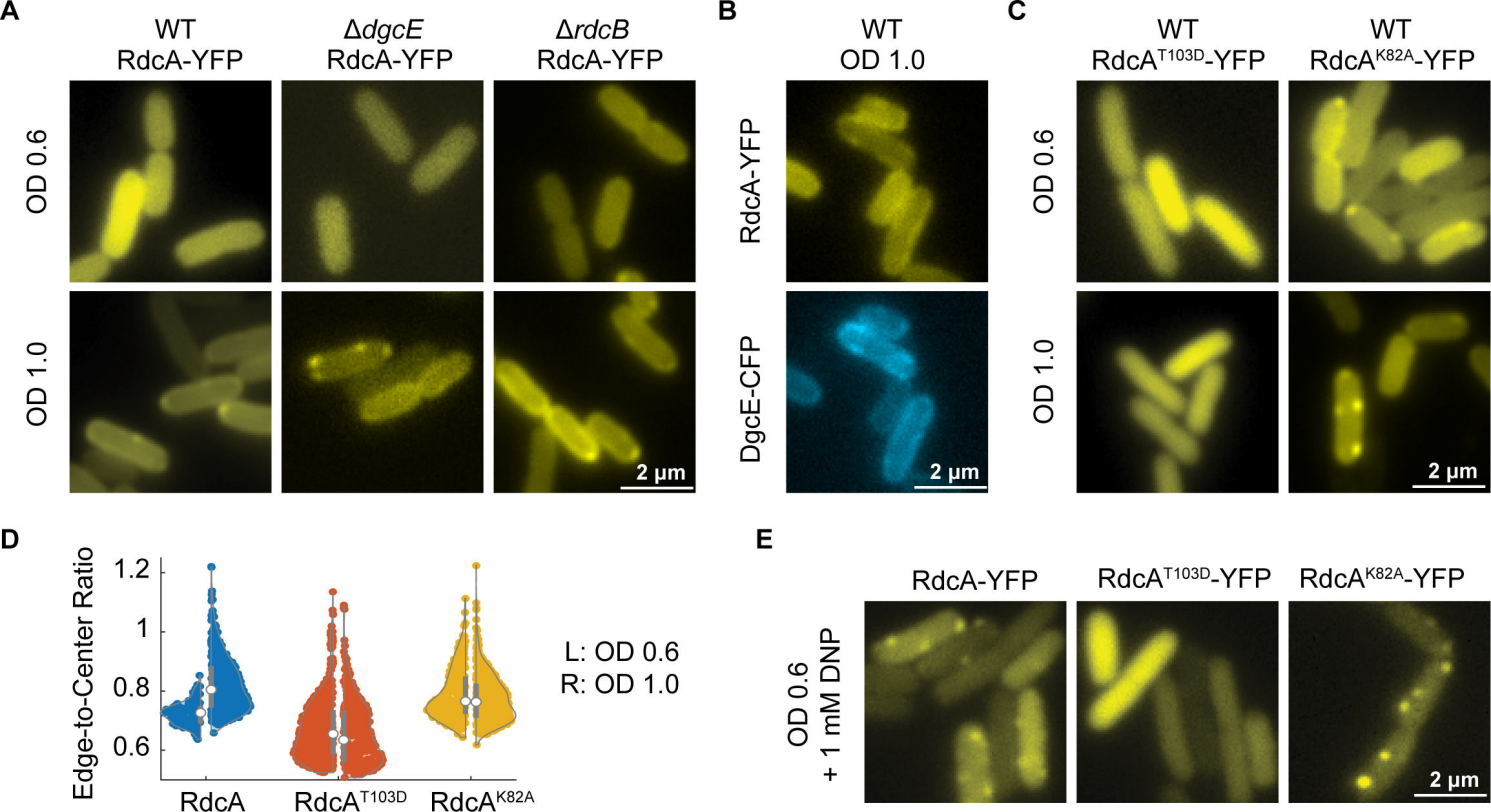
Dependence of RdcA localization on growth phase, GTP binding, and cell energy state. (**A**) Localization of RdcA-YFP in WT, ∆*dgcE,* and ∆*rdcB* cells. Cells were cultivated until the indicated optical density (OD) at 600 nm and immobilized on agarose pads for fluorescence microscopy. (**B**) Fluorescence microscopy images of wild-type cells coexpressing DgcE-CFP and RdcA-YFP. (**C**) Fluorescence microscopy images illustrating the localization of C-terminally YFP-labeled RdcA^T103D^ (GTP-bound) or RdcA^K82A^ (GTP-free) variants. (**D**) Quantification of cellular distribution of RdcA and its variants RdcA^T103D^ and RdcA^K82A^. “Edge-to-center ratio” is defined as the ratio of cell edge-associated fluorescence intensity to intracellular intensity. Data represent single-cell measurements and the median (white dot) at an OD of 0.6 (left) and 1.0 (right) in a representative experiment. Interquartile range is indicated by the thick gray lines. (**E**) Distribution of RdcA variants in cells harvested at an OD of 0.6 and imaged under an agarose pad with 1 mM DNP (energy-depleting condition). All microscopy images are representative of at least three biological replicates. Growth media were supplemented with 30 µM IPTG for RdcA-YFP expression and, in panel **B**, additionally with 0.02% L-arabinose for DgcE-CFP expression.

Although RdcA lacks a canonical membrane-binding domain, its interaction with the MASE1 domain of DgcE (15) might require recruitment to the cell membrane. Indeed, although fluorescently labeled RdcA-YFP was uniformly distributed across the *E. coli* cytoplasm at exponential phase (OD 0.6), it became enriched at the cell membrane upon entry to early stationary phase (OD 1.0) (Fig. 2A). This difference correlated with the growth-phase dependence of the interaction between RdcA and DgcE (Fig. 1E), suggesting that the two may be related. We further observed the formation of membrane-localized polar and subpolar RdcA-YFP foci, as well as smaller foci distributed along the cell body (Fig. 2A). Polar and subpolar foci were also occasionally detected at native expression levels of RdcA-YFP from the original chromosomal locus (Fig. S2), although their size was smaller. In contrast, the overall membrane staining and lateral foci were not readily detectable at the native expression level, likely due to low signal intensity.

When DgcE-CFP was coexpressed with RdcA-YFP, as in FRET experiments, both proteins colocalized to such foci (Fig. 2B), consistent with their interaction. However, recruitment of RdcA to the membrane was equally pronounced in the ∆*dgcE* background, suggesting that it is not a consequence of DgcE binding (Fig. 2A). The presence of RdcB was not required for the membrane localization of RdcA.

The growth phase-dependent localization pattern of RdcA raised the question of whether it may be regulated by GTP binding. Indeed, the constitutively GTP-bound mutant RdcA^T103D^-YFP remained cytosolic, whereas the GTP-free variant RdcA^K82A^-YFP localized to the membrane and formed membrane-localized foci, regardless of growth phase (Fig. 2C). Similar to the wild-type RdcA-YFP, RdcA^K82A^-YFP did not require DgcE or RdcB for localization (Fig. S3A) but colocalized with DgcE-CFP when the two were coexpressed (Fig. S3B). Notably, although the membrane association of the wild-type RdcA increased at the later stage of growth, it remained constantly low for RdcA^T103D^ and constitutively high for RdcA^K82A^ (Fig. 2D; Fig. S3C), supporting our conclusion that RdcA membrane recruitment is regulated by GTP binding and hydrolysis. Finally, membrane localization of the wild-type RdcA, but not of RdcA^T103D^, could be induced in exponentially grown cells by treatment with DNP, resulting in the formation of pronounced membrane-associated foci (Fig. 2E). Again, RdcA and DgcE colocalized to these foci when coexpressed (Fig. S3D). The localization of RdcA^K82A^ was hardly affected by the DNP treatment, although it might have further enhanced foci formation. Overall, this pattern of RdcA membrane recruitment was very similar to the pattern observed for its interaction with DgcE (Fig. 1E). We therefore propose that reduced GTP levels during transition into early stationary phase allow GTP-free RdcA to relocate to the cell membrane, where it interacts with and activates DgcE.

### RdcA and RdcB form a complex independent of growth phase

**Fig 3:**
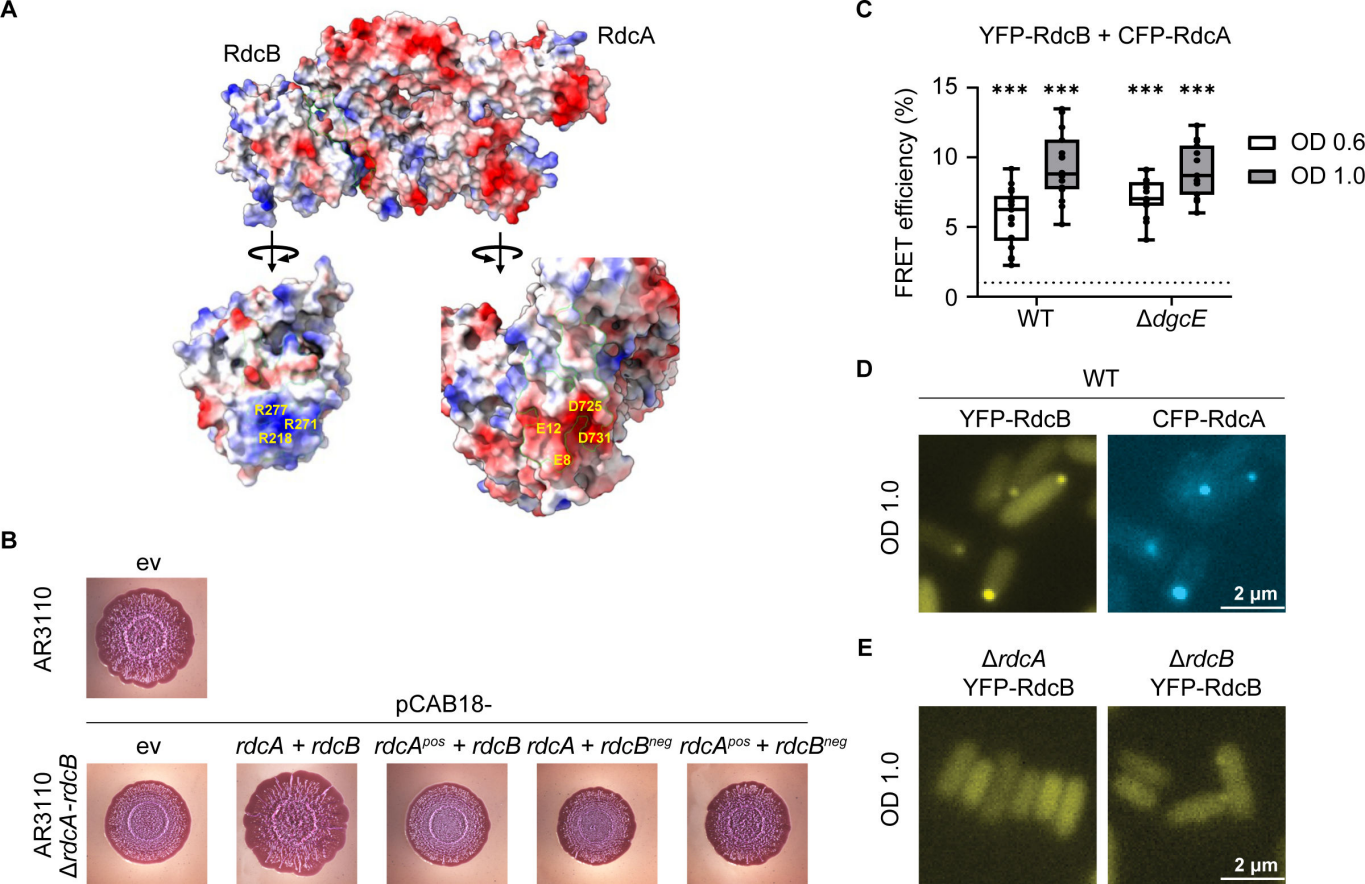
Characterization of the RdcA-RdcB complex. (**A**) Surface charge of the predicted RdcA-RdcB heterodimer model (AlphaFold3), with positive (blue) and negative (red) charges highlighted and residues on the interaction interface labeled. (**B**) Macrocolony formation by the wild-type strain and by the ∆*rdcA-rdcB* deletion strain complemented with indicated wild-type or mutant genes on Congo red plates for 5 days at 28°C. (**C**) Interaction between YFP-RdcB and CFP-RdcA was determined by acceptor photobleaching-based FRET in either wild-type or ∆*dgcE* background. Each data point represents a technical replicate, measured in three biological replicates. Statistical significance was determined by a one-tailed *t*-test against 1% (threshold for significant interaction; dashed line) (***P < 0.0005). (**D**) Fluorescence microscopy images demonstrating the colocalization of YFP-RdcB and CFP-RdcA in wild-type cells collected at an OD of 1.0. (**D and E**) YFP-RdcB and CFP-RdcA expression was induced by 20 µM IPTG and 0.01% L-arabinose, respectively. (**E**) Localization of YFP-RdcB in ∆*rdcA* (left) and ∆*rdcB* cells (right) collected at an OD of 1.0. To ensure low YFP-RdcB expression levels from the leaky pTrc99a promoter, no IPTG was added.

While RdcB is required for RdcA to activate DgcE (15), its mechanistic role has remained unclear. Therefore, we next investigated the complex formation between RdcA and RdcB. The RdcA-RdcB heterodimer predicted using AlphaFold (29) involves electrostatic interactions mediated by several negatively charged amino acids at the N-terminal and the C-terminal helices of RdcA and positively charged amino acids at the C-terminal helix, a β-sheet, and an unstructured loop of RdcB (Fig. 3A). The importance of these charged amino acids was tested by replacing the negatively charged residues in RdcA with arginines (*rdcA^pos^*) and positively charged arginines in RdcB with glutamates (*rdcB^neg^*). When coexpressed with the native partner protein, these charge-reversal variants failed to exhibit interactions in the BACTH assay (Fig. S4A) or to rescue the phenotype of the Δ*rdcA-rdcB* strain in macrocolony assays (Fig. 3B). However, coexpression of both charge-reversal variants (*rdcA^pos^* + *rdcB^neg^*) could partially restore wrinkled macrocolony morphology, indicating that these proteins can interact, although their interaction was not strong enough to be detected by the BACTH assay (Fig. S4A).

We further tested the growth-phase dependence of the formation of the RdcA-RdcB complex. Although degradation of RdcA was previously reported (15), we observed that chromosomally FLAG-tagged RdcA and RdcB were present as full-length proteins throughout growth phases (Fig. S4B). Both proteins formed a complex already during exponential growth, and this complex formation was independent of DgcE (Fig. 3C). When coexpressed, RdcA and RdcB fluorescent protein fusions colocalized in large membrane foci throughout growth (Fig. 3D; Fig. S4C). RdcB was cytosolic in the absence of RdcA but was recruited to the membrane already at native expression levels of RdcA (Fig. 3E; Fig. S4D). Taken together, we conclude that RdcA and RdcB form a constitutive complex through electrostatic interactions, resulting in the membrane recruitment of RdcB by RdcA.

### RdcB is required for assembly and functionality of the RdcA-RdcB-DgcE complexes

**Fig 4:**
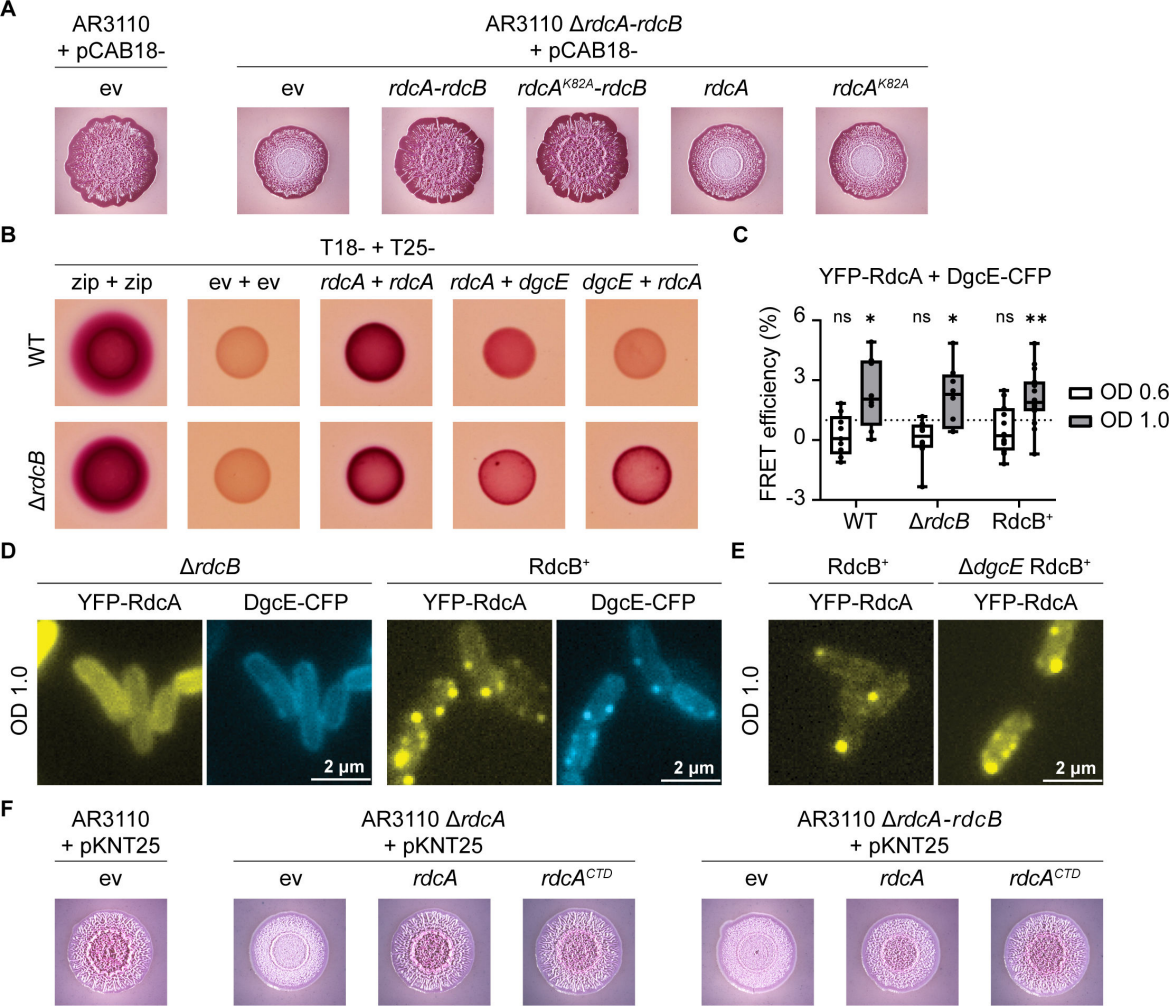
Role of RdcB in assembly and activity of the RdcA-RdcB-DgcE complex. (**A**) Macrocolony formation by AR3110 wild-type or ∆*rdcA-rdcB* strains complemented with indicated constructs on Congo red plates for five days at 28°C. (**B**) BACTH-based analysis of interaction between RdcA and DgcE in wild-type (WT) and ∆*rdcB* backgrounds. (**C**) FRET analysis of interaction between YFP-RdcA and DgcE-CFP at native *rdcB* expression (WT), in the absence of *rdcB* (∆*rdcB*), and under *rdcB* overexpression (RdcB^+^). Data are represented as boxplots, where each data point is a technical replicate measured in three biological replicates. Statistical significance was determined by one-tailed *t*-test against 1% (threshold for significant interaction; dashed line) (*P < 0.05, **P < 0.005; “ns,” not significant). (**D**) Fluorescence microscopy images showing colocalization of YFP-RdcA and DgcE-CFP expressed in ∆*rdcB* or RdcB^+^ strains. (**E**) Localization of RdcA-YFP coexpressed with RdcB (RdcB^+^) in the presence or absence of *dgcE*. (**C–E**) YFP-RdcA and DgcE-CFP were expressed from pTrc99a and pBAD33 plasmids, respectively, induced with 50 µM IPTG and 0.02% L-arabinose. (**F**) Macrocolony morphology of wild-type, ∆*rdcA,* or ∆*rdcA-rdcB* strains complemented with the pKNT25 plasmid expressing full-length RdcA or its CTD.

We next aimed to elucidate the function of RdcB in the regulation of DgcE by RdcA. One possible function of RdcB could be to promote the GTP hydrolysis by RdcA. However, we observed that the constitutively active RdcA^K82A^ variant, which does not bind GTP at all, failed to rescue a Δ*rdcA-rdcB* mutant unless *rdcB* was coexpressed, suggesting that RdcB is required for DgcE activation independently of GTP regulation (Fig. 4A). Furthermore, the functional requirement for RdcB was not due to RdcA stabilization, since RdcA protein levels were unaltered by *rdcB* deletion (Fig. S5A). Furthermore, RdcB was neither required for RdcA dimerization nor for the interaction between RdcA and DgcE (Fig. 4B and C).

However, we observed that coexpression of RdcB (RdcB^+^) along with YFP-RdcA from the same operon largely enhanced the spatial organization of the RdcA-DgcE complex into discrete foci, as compared to the Δ*rdcB* or the wild-type background (Fig. 4D; Fig. S5B). RdcB-induced RdcA foci formation persisted in the Δ*dgcE* background (Fig. 4E). These data suggest that RdcB promotes membrane-located cluster formation by the wild-type RdcA, independently of DgcE. In contrast, the RdcA^K82A^ variant formed large clusters even in the absence of RdcB (Fig. S3A).

We further observed that RdcA^CTD^, the domain responsible for DgcE interaction and activation, restored macrocolony biofilm formation to a similar extent in both Δ*rdcA* and Δ*rdcA-rdcB* backgrounds (Fig. 4F). In contrast, the full-length RdcA showed much reduced activity in the Δ*rdcA-rdcB* backgrounds. This finding indicates that RdcB might induce a more open active conformation of the full-length RdcA, a regulatory requirement that is bypassed by RdcA^CTD^.

### (p)ppGpp modulates the RdcA-RdcB-DgcE signaling pathway

**Fig 5:**
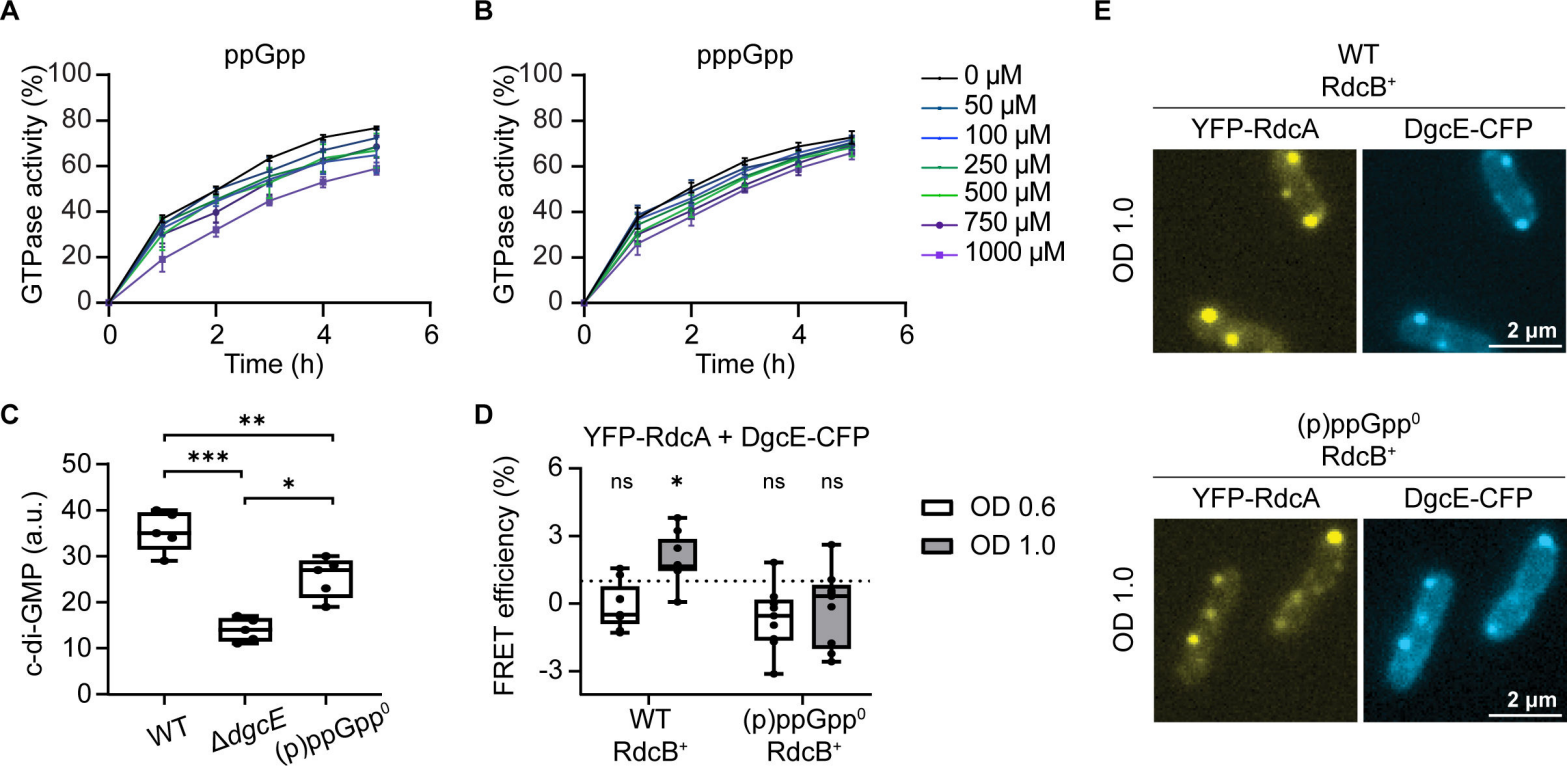
(p)ppGpp modulates RdcA-RdcB-DgcE signaling. (**A and B**) The GTPase activity of RdcA is inhibited by (p)ppGpp. Hydrolysis of [α-^32^P]-GTP (supplemented with 100 µM unlabeled GTP) was monitored over time in the presence of indicated concentrations of either ppGpp (**A**) or pppGpp (**B**). Reaction products were separated by thin-layer chromatography and quantified using ImageJ. Data represent the mean ± SD (*n* = 3). (**C**) Relative c-di-GMP levels inferred using a FRET biosensor of c-di-GMP in either the wild-type (WT) strain, ∆*dgcE*, or in ∆*relA* ∆*spoT* strain ((p)ppGpp^0^). Biosensor was expressed from the pTrc99a plasmid, supplemented with 100 µM IPTG for induction. Data are presented as mean ± SD, with each data point from a biological replicate (*n* = 3–5). Statistical significance was determined by paired *t*-tests, with *P < 0.05, **P < 0.005, ***P < 0.0005. (**D**) Effect of (p)ppGpp on FRET between YFP-RdcA and DgcE-CFP. Measurements were performed in strains coexpressing RdcA with RdcB (RdcB^+^) to ensure proper RdcA:RdcB stoichiometry. Data are represented by boxplots, where each data point represents a technical replicate from three biological replicates. Statistical significance was determined by a one-tailed *t*-test against 1% (dashed line) (*P < 0.05; “ns,” not significant). (**E**) Fluorescence microscopy images showing colocalization of YFP-RdcA and DgcE-CFP, with RdcB being coexpressed with RdcA (RdcB^+^), in a wild-type or (p)ppGpp^0^ background. Cells were collected at OD 1.0. (**C–E**) Media were supplemented with 50 µM IPTG and 0.02% L-arabinose for induction.

Since the alarmone (p)ppGpp can competitively bind and inhibit P-loop GTPases (30–32), we tested whether (p)ppGpp might also affect RdcA activity. We indeed observed that both ppGpp and pppGpp reduced GTP hydrolysis by RdcA in a dose-dependent manner (Fig. 5A and B). A 15%–30% inhibition occurred at a 10:1 ratio of (p)ppGpp to GTP (1 mM vs. 100 µM), which is physiologically relevant during the stationary phase (33).

To assess the significance of this inhibition, we measured cellular c-di-GMP levels in the *E. coli* (p)ppGpp^0^ strain that lacks both the (p)ppGpp synthase RelA and the bifunctional enzyme SpoT that both synthesizes and degrades (p)ppGpp. The measurements were performed using a recently described FRET-based c-di-GMP biosensor that consists of a c-di-GMP-binding YcgR homolog from *Thermincola potens* fused to the FRET pair mNeonGreen and mTurquoise2 (34). The absence of (p)ppGpp resulted in significantly lower c-di-GMP levels (Fig. 5C), supporting the idea that (p)ppGpp competes with GTP to alleviate the inhibition of RdcA activity. However, c-di-GMP levels in the (p)ppGpp^0^ strain remained higher than those in Δ*dgcE*, suggesting that the (p)ppGpp-dependent regulation is not essential for activation but rather acts as an auxiliary mechanism to fine-tune c-di-GMP levels during growth-phase transitions.

Finally, we examined the impact of (p)ppGpp on the RdcA-RdcB-DgcE complex. Under coexpression of RdcB (RdcB^+^), the RdcA-DgcE FRET signal was significantly reduced in early stationary-phase cells in the absence of (p)ppGpp (Fig. 5D). However, the absence of (p)ppGpp did not apparently alter the colocalization RdcA-DgcE to membrane foci (Fig. 5E). We therefore propose that (p)ppGpp might fine-tune DgcE activity by inducing structural rearrangement within the RdcA-RdcB-DgcE complexes without disrupting their assembly.

## DISCUSSION

As a central regulator of biofilm formation by *E. coli*, c-di-GMP is tightly controlled across growth stages. During the exponential and post-exponential phases, c-di-GMP levels remain low, permitting flagellar motility and thereby facilitating resource foraging (14, 35). Upon entry into the stationary phase, growth is reduced in favor of stress survival functions under the control of the general stress response sigma factor RpoS. This includes an increase in c-di-GMP levels to promote the formation of biofilms, which also provide protection against various environmental stresses, including predation (36, 37). As characterized in detail in the present study, this growth phase-dependent transition from low to high c-di-GMP levels in *E. coli* relies not solely on the increased expression of RpoS-controlled DGCs (9, 14) but also on the post-translational activation of c-di-GMP synthesis by DgcE, mediated by the dynamin-like sensory GTPase RdcA.

**Fig 6:**
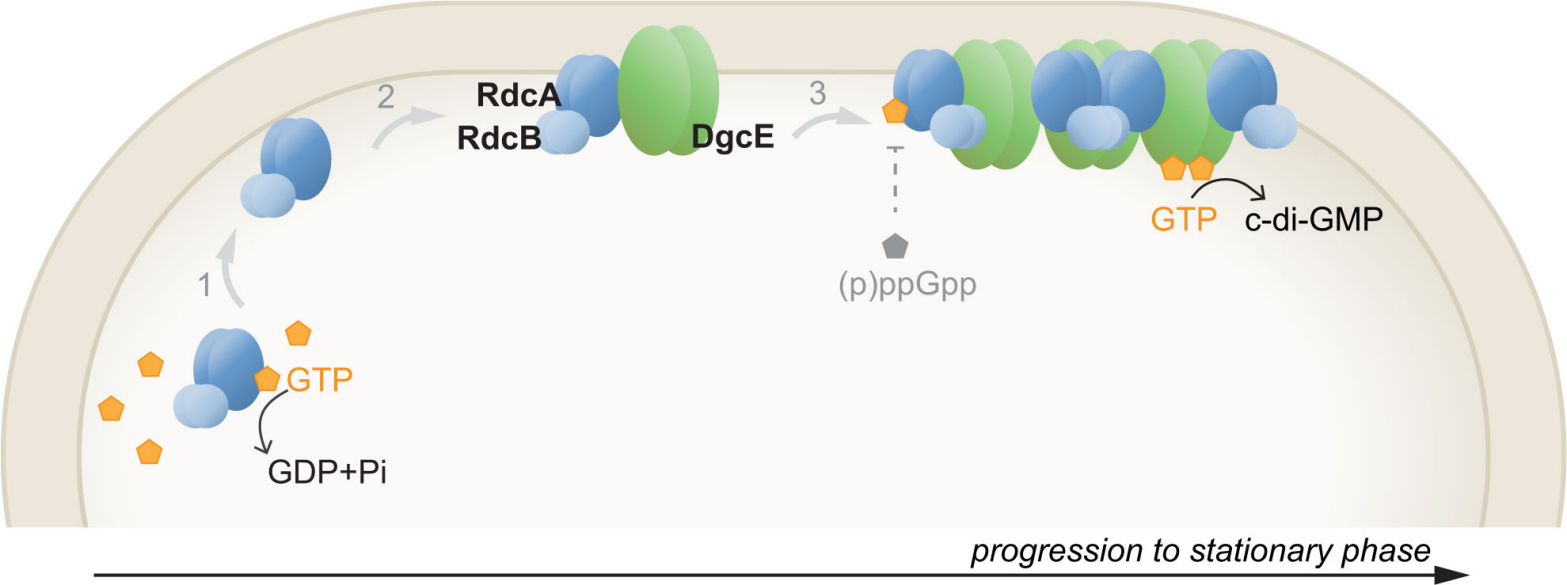
Schematic model of the proposed regulation of c-di-GMP production by the RdcA-RdcB-DgcE complex. During the exponential growth phase (left), high intracellular GTP levels retain RdcA predominantly in its GTP-bound state, preventing its recruitment to the cytoplasmic membrane and interaction with DgcE. During progression to the stationary phase (from left to right), reduced GTP levels permit membrane localization of RdcA (1), thereby promoting its binding to DgcE (2). RdcB, which forms a stable complex with RdcA throughout growth, is recruited to the cell membrane along with RdcA and promotes the ternary complex assembly and activation of DgcE (3). Elevated (p)ppGpp levels may further indirectly modulate the pathway by competing with GTP for RdcA binding.

Our data indicate that RdcA functions as a GTP-sensitive switch, activating DgcE in response to declining cellular GTP levels, which can occur during growth phase transitions due to amino acid starvation and/or to reduced cellular energy status (Fig. 6). During the post-exponential growth phase, RdcA and RdcB are already coexpressed with flagella under FliA control (38), while the production of DgcE is largely RpoS-dependent and occurs slightly later (9, 14). However, high GTP levels in the still metabolically active and fully energized cells maintain RdcA in its GTP-bound form, preventing its membrane association and thereby ensuring low c-di-GMP levels. Subsequently, upon transition into the stationary phase, or possibly also when cells are deenergized due to other reasons, GTP levels drop, thereby relieving inhibition of RdcA by bound GTP and enabling it to localize to the cell membrane, where it activates DgcE-mediated c-di-GMP synthesis. The physiological relevance of this regulatory mechanism is underscored by RdcA’s low GTP affinity, with a *K_m_* ~0.6 mM falling into the range of GTP concentrations between exponential (~ 1 mM) and stationary (0.1–0.2 mM) phases (24, 25). However, the inhibition of RdcA by GTP seems more complex than the classical allosteric control by reversible ligand binding because the GTPase-inactive variant RdcA^T103D^ remains fully locked in the inactive state even in stationary phase, where GTP levels are low. In other words, the enzymatic GTPase activity of RdcA is apparently required for RdcA to actively free itself of its inhibitor GTP in order to associate with the membrane and activate DgcE. Thus, RdcA can be viewed as a “trigger enzyme” (39, 40) that uses its enzymatic function as a sensory input for controlling the activity of its interaction partner. In fact, trigger enzymes are a recurring theme in c-di-GMP signaling in *E. coli* (39, 41, 42).

Beyond sensing GTP, RdcA appears to respond to the alarmone (p)ppGpp, which plays complex and multiple roles during the transition into stationary phase (43). During the stringent response, a consequence of amino acid limitation, accumulation of (p)ppGpp leads to a global adjustment of protein biosynthesis, including a strong reduction in ribosome production, and subsequently to the replacement of the vegetative sigma factor (RpoD) with RpoS. Our data suggest that this accumulation of (p)ppGpp may also contribute to DgcE activation by competitive binding to RdcA. Indeed, RdcA-mediated GTP hydrolysis was partially inhibited when (p)ppGpp levels exceeded GTP levels by a factor of 10, which is within the physiological range when *E. coli* cells enter the stationary phase (33). This is consistent with previous reports that several P-loop GTPases are inhibited by (p)ppGpp, which shares a similar structure with GTP and competes for the same active center (30–32). Nevertheless, this regulation by (p)ppGpp apparently only plays an auxiliary role for the RdcA-mediated activation of DgcE.

We thus propose that, by responding to GTP and (p)ppGpp levels, RdcA couples the cellular metabolic and energy status to the decision to initiate biofilm formation via its post-translational control of DgcE. This is an intriguing example of a finely tuned regulatory cascade, which combines the post-exponentially deployed RdcA/RdcB with the later-produced DgcE to sense the shift in nucleotide ratio caused by declining GTP and rising (p)ppGpp levels. The resulting increase in c-di-GMP triggers the PdeR/DgcM/MlrA pathway, leading to expression of the curli transcription factor CsgD, which is required for extracellular matrix production (36). The need for such precise tuning of this complex cascade of events likely reflects the tradeoff between a protective function of the biofilm matrix and the high cost of its production. This means that cells must initiate biosynthesis of these polymers already at a certain threshold of resource limitation, prior to the onset of starvation that would no longer allow them to produce the matrix polymers.

Although GTP reduction and (p)ppGpp accumulation can signal amino acid limitation, reduced cellular energy can also directly lead to GTP depletion, thus enabling RdcA to integrate multiple cues through GTP sensing. The coupling between the GTP pool and RdcA-dependent c-di-GMP production likely explains the recently described link between membrane potential and c-di-GMP levels (34). We hypothesize that an interplay between GTP levels and c-di-GMP signaling could be widespread among bacteria, although specific mechanisms might differ. For instance, GTP was shown to activate a PDE in *Caulobacter crescentus*, which could similarly maintain low c-di-GMP levels under high GTP conditions (44).

Although the detailed molecular mechanisms of this regulation of DgcE activity remain to be fully elucidated, we could at least partly characterize the mechanism of RdcA function. RdcA possesses a modular structure consisting of an N-terminal GTPase domain and a C-terminal domain, which have distinct but integrated roles. While the C-terminal domain is responsible for the interaction and activation of DgcE, the N-terminal GTPase domain confers the regulatory function. How RdcA is recruited to the membrane, which also occurs in the absence of DgcE, remains to be unraveled. The role of RdcB remains less clear, but our data indicate that it interacts with both the C-terminal and the N-terminal domains of RdcA. RdcB does not seem to be required for either membrane localization of RdcA, its interaction with DgcE, or DgcE dimerization. We therefore hypothesize that the interaction with RdcB might promote a structural rearrangement within RdcA and/or the assembly of larger RdcA-RdcB-DgcE complexes that may be required for DgcE activation.

Our study thus reveals a unique role for the bacterial dynamin homolog RdcA as a GTP/(p)ppGpp sensor that feeds into signaling by the second messenger c-di-GMP to control the inverse growth vs. survival balance (36). This novel type of sensory GTPase expands the spectrum of GTPases, such as small Ras-like GTPases, that are common in eukaryotic and bacterial signaling pathways (45).

## MATERIALS AND METHODS

### Bacterial strains

All *in vivo* experiments and measurements in this study were performed in the derivatives of *E. coli* K-12 strain W3110 with functional RpoS (W3110 *rpoS*+) (11) or AR3110, a derivative of W3110 with functional BcsQ (11) (Table S1). Gene deletions were generated using the one-step inactivation method, as described previously (46). Briefly, chromosomal genes are replaced by antibiotic resistance cassettes with overhangs homologous to the sequences flanking target genes based on λ Red technology. Primers used for homologous recombination are listed in Table S2. PCR products containing kanamycin resistance cassettes were either amplified from Keio collection strains or pKD13 (47). The resistance cassettes, as well as the plasmid used to induce λ Red expression, were removed for later experiments if necessary. A transfer of mutations was performed via P1 transduction. A modified version of the one-step inactivation was used to introduce chromosomal 3× FLAG tags (48). The plasmid used for the PCR amplification of the selectable marker additionally carries the 3× FLAG sequence. Using primers with homologous extensions to the downstream sequence of the target gene allows integration by λ Red-mediated homologous recombination. Strains containing chromosomal amino acid substitutions (*rdcA^K82A^* and *rdcA^T103D^*) were constructed before (15, 26). *E. coli* BL21 (DE3) strain was used to overexpress and purify the RdcA-His_6_ protein.

### Growth conditions

For FRET measurements or microscopy, bacterial cultures were grown in liquid tryptone broth (TB) medium at 30°C, with shaking at 220 revolutions per minute (rpm). Growth was followed by determining the optical density at 600 nm. For immunoblot, the cells were cultivated in lysogeny broth (LB) medium at 28°C under shaking conditions, and the optical density was determined at 578 nm. For strains carrying plasmids, 100 µg/mL ampicillin, 50 µg/mL kanamycin, and/or 34 µg/mL chloramphenicol were supplemented.

Macrocolony biofilms were grown as described before (11). In short, 5 µL of an overnight culture grown at 37°C (free of matrix) was spotted on LB agar plates without salt and was incubated for 5 days at 28°C. The addition of Congo red (40 µg/mL) and Coomassie brilliant blue (20 µg/mL) allowed the staining of the extracellular matrix components curli and cellulose. Pictures were taken with a Stemi 2000-C stereomicroscope (Zeiss) coupled to an AxioCamICC3 camera and with 10-fold magnification.

### Construction of plasmids and gene mutagenesis

Plasmids (Table S3) were constructed based on restriction enzyme cloning or Gibson assembly with primers listed in Table S4. Genes of interest were amplified from colonies of W3110 strains. For the fluorescence microscopy, genes were tagged with fluorophores at N-/C-termini, as indicated, through a flexible linker. Mutagenesis of *rdcA* (*rdcA^T103D^* and *rdcA^K82A^*) was generated using designed primers, and mutated alleles were confirmed by sequencing (as previously described by Pfiffer et al. [15]).

### Bacterial two-hybrid assay

To investigate *in vivo* protein-protein interactions using the adenylate cyclase-based bacterial two-hybrid system (15, 22), sequences of target genes were cloned on the pUT18/pUT18C and pKT25/pKNT25 plasmids as described above to create N- or C-terminal CyaA protein fusions. *E. coli* W3110 Δ*cyaA* was co-transformed with the different plasmid combinations and incubated overnight on selective MacConkey agar plates. Single colonies were resuspended in 50 µL ddH_2_O and spotted on fresh MacConkey agar plates containing antibiotics and 1% lactose. The colonies were inspected, and images were taken after 24 h of incubation at 30°C. The leucine zipper domain of yeast’s GCN4 transcription factor was used as a positive control, and an empty vector was used as a negative control.

### Acceptor photobleaching FRET assay

FRET measurements by acceptor photobleaching were conducted as described previously (28). For FRET measurement, the cells were cultivated by inoculating 100 µL overnight culture into 10 mL fresh TB medium supplemented with indicated concentrations of isopropyl-β-D-thiogalactoside (IPTG) and L-arabinose (see figure legends). When OD_600_ reached ~0.6/~1.0, 1 mL of the cell culture was harvested by centrifugation at 4,000 × *g* for 5 min, washed with tethering buffer (10 mM KH_2_PO_4_, 10 mM K_2_HPO_4_, 0.1 mM EDTA, 1 μM L-methionine, and 10 mM sodium lactate, pH 7.0), and concentrated 50-fold. Cells were loaded onto a microscope glass slide and immobilized under a 100 µL agarose pad (1% agarose in tethering buffer) and a coverslip. For DNP treatment, the agarose pad was prepared with 1 mM DNP.

For each biological replicate, images were acquired at three isolated positions as technical replicates on a Nikon Ti microscope with a 40×/1.3 NA oil objective and an Andor Ixon 897-X3 EM-CCD camera (Andor Technology), controlled by NIS-Elements AR software (v4.40; Nikon). Images were acquired with 1 s exposure in a time sequence in two spectral channels: (i) 2 images in the acceptor channel (YFP), followed by (ii) 120 images in the donor channel taken before the photobleaching, then by (iii) 4 s acceptor photobleaching using a 532 nm laser with 40% power (no image acquisition), (iv) 40 images in the donor channel, and (v) 2 images in the acceptor channel. Images were analyzed using ImageJ software (49). The fluorescence background intensity was subtracted from the signal. FRET efficiency was obtained by calculating the percentage of donor signal increase (CFP) after photobleaching, as described previously, with signal increase above 1% indicating positive protein interaction (28). A linear fit (RStudio) was applied to correct for the bleaching of the donor due to light exposure.

### Fluorescence microscopy imaging

To prepare a sample for imaging analysis, cells were grown as described for the FRET experiments above. When OD_600_ reached ~0.6/~1.0, 3 µL of the cell culture was loaded onto a glass slide and covered with a 400 µL 1% agarose pad and a coverslip. Samples were mounted on a Nikon Ti microscope with a 100×/1.45 NA oil objective, equipped with a perfect focus system. Images were acquired by a sCMOS camera (Andor, Zyla 4.2) in corresponding spectral channels.

For membrane localization analysis, the cell boundary was determined with the Omnipose segmentation algorithm (50), which was executed based on the phase-contrast images. The resulting segmented masks were then used to determine the fluorescence intensity at the cell membrane (edge) and in the intracellular region. The mean of edge-associated pixel intensity was divided by the mean intracellular pixel intensity to give an estimation of the ratio of membrane- to cytosolic-localized protein in individual cells. A comparative analysis of the data obtained at an OD of 0.6 and 1.0 was performed by bootstrapping the sample median.

### SDS polyacrylamide gel electrophoresis and immunoblot detection

Cultures for immunoblot detection were grown at 28°C in LB medium under shaking conditions, and samples were taken at the indicated time points. Similar amounts of total cellular protein were taken, resuspended in SDS-PAGE sample buffer, and incubated for 10 min at 100°C. After similar amounts of total protein were loaded along with the WesternSure Pre-stained Chemiluminescent Protein ladder (Li-Cor), proteins were separated in an SDS polyacrylamide gel electrophoresis; 3× FLAG-tagged proteins were specifically detected by immunoblotting using a 3× FLAG-tag antibody (1:10,000, produced in mice), followed by an anti-mouse IgG horseradish peroxidase conjugate (1:300,000, GE Healthcare) antibody. Chemiluminescence was visualized by adding Western Lightning Plus-ECL and a CCD camera (ImageQuant LAS4000).

### Protein purification

RdcA was cloned on the multiple cloning site 1 (MCS1) of the pETDuet-1 vector fused to a His_6_-tag. *E. coli* BL21 (DE3) was transformed with the plasmid, grown in LB at 37°C, and T7-dependent expression was induced at OD 0.6 with 100 mM IPTG. Expression was carried out overnight at 16°C. Cells were harvested by centrifugation, and pellets were resuspended in lysis buffer (20 mM HEPES, pH 7, 300 mM KCl, 10 mM MgCl_2_) containing lysozyme, protease inhibitor cocktail (complete, EDTA-free; Roche), and DNase 1. Cells were lysed with a French Press (3×, 18,000 psi), and the membrane fraction was isolated by centrifugation at 7,000 rpm for 20 min and resuspended in solubilization buffer (20 mM HEPES, pH 7, 300 mM KCl, 10 mM MgCl_2_, 2% dodecyl-β-D-maltoside [DDM; Roth]). After solubilization for 2 h at 4°C under gentle shaking conditions, the insolubilized material was removed by ultracentrifugation (60 min at 36,000 rpm). For purification of RdcA-His_6_, the supernatant was loaded on an equilibrated (20 mM HEPES, pH 7, 300 mM KCl, 10 mM MgCl_2_; 20 mM imidazole) Ni-NTA column using the Äkta purifier and eluted with 250 mM imidazole. For higher purity, the flowthrough was reloaded on the Ni-NTA. Since the RdcA protein tends to polymerize or precipitate, it was unsuitable for size exclusion chromatography or concentration with centrifugal filters. Therefore, the elution fractions containing RdcA were pooled and concentrated with PEG-6000. Imidazole and PEG were removed by dialysis overnight. Aliquots of the fusion protein were frozen in liquid nitrogen and stored at −80°C. The whole purification process was followed by taking samples and analyzing the RdcA-His_6_ content on an SDS-PAGE gel. The RdcA variants (RdcA^K82A^ and RdcA^T103D^) were purified with the same protocol.

### Protein activity assay/GTPase assay

The *in vitro* GTPase activity of RdcA-His_6_ was measured with two different methods. The colorimetric malachite green assay allows the determination of the concentration of inorganic phosphate produced during GTP hydrolysis as a byproduct. The molybdate contained in the malachite green reagent forms a complex with P_i_, which is bound by malachite green and results in a green color. Since its intensity is directly proportional to the Pi concentration, the spectrophotometric measurement at 620 nm enables the calculation of phosphate in the used reaction buffer (20 mM HEPES pH 7, 300 mM KCl, 10 mM MgCl_2_) with a standard curve of defined phosphate concentrations; 5 µM of RdcA-His_6_ was mixed with varying GTP concentrations and incubated for 1 h at 37°C. Data were normalized to the amount of protein added. GraphPad Prism was used to determine the Michaelis-Menten kinetics and calculate the dynamic values.

Since the available purified ppGpp and pppGpp (JenaAnalytics) contain phosphate as a byproduct, the malachite green assay was not suitable to investigate the effect of (p)ppGpp on the enzymatic activity. For that reason, the hydrolysis in the presence of varying ppGpp and pppGpp concentrations was followed over time by incubating RdcA-His_6_ with a mix of unlabeled GTP (100 µM) and 8 nM α-^32^P-GTP (Hartman Analytic GmbH). The products were separated by thin-layer chromatography and quantified with ImageJ.

### c-di-GMP measurement using flow cytometry-based FRET

The c-di-GMP sensor was constructed as described previously (34) by tagging a YcgR homolog from *Thermincola potens* with mNeonGreen and mTurquoise2 fluorophores at the N- and C-termini, respectively, expressed from an IPTG-inducible vector pTrc99a. The flow cytometry-based FRET assay was performed using a BD LSRFortessa SORP cell analyzer (BD Biosciences, Germany). Fluorescence intensities in the donor emission (I_1_, 470/15-nm filter) and FRET (I_2_, 520/35-nm filter) channels were acquired under donor (mTurquoise2) excitation at 445 nm, and fluorescence intensity in the acceptor emission (I_3_, 520/30-nm filter) channel was acquired under acceptor (mNeonGreen) excitation at 488 nm. Flow cytometry data were analyzed using FlowJo (BD Biosciences), and the values were used to calculate FRET efficiencies as described before (34).

## Data Availability

All data and materials are available upon request.

## SUPPLEMENTAL MATERIAL

Supplemental Figures mbio.00668-26-s0001.docx Fig. S1 to S5.

Supplemental Tables mbio.00668-26-s0002.docx Tables S1 to S4.

ASM does not own the copyrights to Supplemental Material that may be linked to, or accessed through, an article. The authors have granted ASM a non-exclusive, world-wide license to publish the Supplemental Material files. Please contact the corresponding author directly for reuse.
