# Supplementary material for: Bacterial dynamin homolog regulates growth phase-dependent c-di-GMP production
Source: mBio. 2026 Jul 20;17(8):e00668-26. doi: 10.1128/mbio.00668-26 (PMC13463826; doi:10.1128/mbio.00668-26)
Supplement: Supplementary file 1 [file mbio.00668-26-s0001.docx]

**SUPPLEMENTAL FIGURES**

**Figure S1. RdcA showed enhanced interaction with DgcE at early stationary phase of growth. (A)** Growth curve of *E. coli* W3110 incubated in TB medium at 30 °C with shaking at 200 rpm. Data represent the mean ± SD (n=6). Samples were collected for acceptor photobleaching FRET assay and subcellular localization imaging at exponential phase (OD ~0.6, 5 h) and early-stationary phase (OD ~1.0, 7 h). **(B)** Interaction between DgcE-CFP and RdcA-YFP or indicated mutants quantified using acceptor photobleaching-based FRET assay. Shown are the same data as in Figure 1E but with additional statistical analysis. Statistical significance was determined by unpaired t-tests (**p* < 0.05, ***p* < 0.005, ****p* < 0.0005; “ns”, not significant).

**Figure S2. Localization of RdcA-YFP expressed from native chromosomal locus.** Fluorescence images showing localization of RdcA-YFP expressed from the native genomic locus genome. Enlarged cells on the right show examples of foci formation (indicated by arrows).

**Figure S3. Localization of RdcA and its variants RdcA^K82A^ and RdcA^T103D^. (A)** Fluorescence microscopy images of ∆*dgcE* and ∆*rdcB* cells expressing RdcA^K82A^-YFP. **(B)** Fluorescence microscopy images of wild-type (WT) cells co-expressing DgcE-CFP and RdcA^K82A^-YFP. **(C)** The magnitude and consistency of changes in “Edge-to-Center Ratio” were determined by bootstrapping the median difference between two groups (OD 1.0 - OD 0.6) for three independent experiments. **(D)** Wild-type cells co-expressing DgcE-CFP and wild-type RdcA-YFP treated with 1 mM DNP. Growth media were supplemented with 30 µM IPTG for RdcA-YFP expression, and where applicable with 0.02% L-arabinose for DgcE-CFP expression.

**Figure S4.** **Interaction, expression and colocalization of RdcA and RdcB. (A)** BACTH-based analysis of interaction between RdcA and RdcB and their charged residue substitution mutants. **(B)** Expression of RdcA and RdcB throughout growth phases. Immunoblot shows detection of RdcA::3xFLAG (~85 kDa) and RdcB::3xFLAG (~35 kDa) at different time points during growth at 28 °C with a specific α-3xFLAG antibody. **(C)** Fluorescence microscopy images demonstrating the colocalization of YFP-RdcB and CFP-RdcA in wild-type (WT) cells collected at OD 0.6. YFP-RdcB and CFP-RdcA expression was induced by 20 µM IPTG and 0.01% L-arabinose, respectively. **(D)** Localization of YFP-RdcB expressed alone in ∆*rdcA* (left) and ∆*rdcB* cells (right), collected at OD 0.6. No additional IPTG was supplemented to maintain low YFP-RdcB expression levels from the leaky pTrc99a promoter.

**Figure S5.** **Stability of RdcA and localization of RdcA and DgcE at native levels of RdcB expression. (A)** Immunoblot showing the detection of RdcA::3xFLAG (~85 kDa) in wild-type (WT) or ∆*rdcB* background with a specific α-3xFLAG antibody. Cultures were grown at 28 °C and samples were taken at OD = 3. **(B)** Fluorescence images of wild-type cells coexpressing YFP-RdcA and DgcE-CFP. Growth medium was supplemented with 50 µM IPTG and 0.02% L-arabinose for induction.
