# Supplementary material for: Bacterial dynamin homolog regulates growth phase-dependent c-di-GMP production
Source: mBio. 2026 Jul 20;17(8):e00668-26. doi: 10.1128/mbio.00668-26 (PMC13463826; doi:10.1128/mbio.00668-26)
Supplement: Supplementary file 2 [file mbio.00668-26-s0002.docx]

Table S1: Bacterial strains used in this study.

| **Name** | **Genotype** | **Reference** |
| --- | --- | --- |
| AR395 | AR3110 Δ*rdcA*::scar | (1) |
| BL21 (DE3) | *fhuA2* [*lon*] *ompT gal* (λ DE3) [dcm] Δ*hsdS* λ  DE3 *=* λ sBamHIo ΔEcoRI-B  *int*::(*lacI*::PlacUV5::T7 gene1) i21 Δ*nin5* | New England Biolabs |
| RON70 | W3110 Δ*cyaA*::scar Δ*rdcB* | this study |
| RON73 | W3110 *rdcB*::3xFLAG::scar | this study |
| RON97 | W3110 *rdcA*::3xFLAG::scar Δ*rdcB* | this study |
| VP51 | AR3110 Δ*rdcA*::scar Δ*dgcE*::scar | (1) |
| VP69 | AR3110 Δ*rdcA-rdcB*::scar | (1) |
| VP551 | W3110 *rdcA*::3xFLAG::scar | (1) |
| W3110 | *E. coli* K-12 *thyA36 deoC2 IN(rrnD-rrnE)I* | (2) |
| VS678 | W3110 Δ*rdcA*::scar | (3) |
| VS679 | W3110 Δ*dgcE*::scar | (3) |
| VS680 | W3110 Δ*rdcB*::scar | (3) |
| VS754 | W3110 *rdcA*::*rdcA-yfp* | (3) |
| XC159 | W3110 Δ*relA*::scar Δ*spoT*::scar | this study |

Table S2: List of primers for strain construction.

| **Description** | **Sequence (5’ – 3’)** |
| --- | --- |
| For *rdcB* deletion | GCGATGATATTCAAACACTTTTCACGGCAGAACGATATTGACCAAGGTGTAGGCTGGAGCTGCTTC |
|  | CACACATGGATTTCGTTTAGGACTCATTGATGTAACTGATTATATTCCGGGGATCCGTCGACC |
| For *rdcB* deletion in *rdcA*::3xFLAG | AGACGTTACTTGACGGCCCCGGTCGCGTGCTGGAGTCGGTTTATGTGTAGGCTGGAGCTGCTTC |
|  | CACACATGGATTTCGTTTAGGACTCATTGATGTAACTGATTATATTCCGGGGATCCGTCGACC |
| For *rdcB*::3xFLAG::scar | GCGTCTGGCGGGGGCAATGCTCGAGGAAATCTTCTCTGTAAATTTGGCGGACTACAAAGACCATGACGG |
|  | GACGGTATTAAGCCAATACAGAACGTACTGATCGACATAATCACAATACCTTAGTTCCTATTCCGAAGTTCCG |
| For *rdcA* deletion | ATCTGGCTAAATAAAATAACAAAATTTGCTTTAAGGAAGAATTTTCTatgATTCCGGGGATCCGTCGACC |
|  | GCGACCGGGGCCGTCAAGTAACGTCTTGGtcaATATCGTTCTGCCGT |
| For *dgcE* deletion | AACACAGAAACGAATACTGGCGACCAGGTCTTGCGGATAAAGCGGTAatgATTCCGGGGATCCGTCGACC |
|  | CGTCGCATCAGGCGATGGGGAAGCACGCCtcaGTTAATCGCGAAATAA  CTTGTAGGCTGGAGCTGCTTCG |
| For *rdcB* deletion | CAGCTGTTACGCGATGATATTCAAACACTTTTCACGGCAGAACGATAttgATTCCGGGGATCCGTCGACC |
|  | GTTTAGGACTCATTGATGTAACTGATTATttaCGCCAAATTTACAGAGAATGTAGGCTGGAGCTGCTTCG |
| For *relA* deletion | CTATCATATGTAGATACAGTATATATCAATCTACATTGT |
|  | ATTTGCCGATTTCGGCAGGT |
| For *spoT* deletion (*kanR* amplified from pKD13) | TTTCGCAGATGCGTGCATAACGTGTTGGGTTCATAAAACAATTCCGGGGATCCGTCGACC |
|  | TATTGCTGAAGGTCGTCGTTAATCACAAAGCGGGTCGCCCTGTAGGCTGGAGCTGCTTCG |

Table S3: List of plasmids used in this study.

| **Name** | **Genotype or phenotype** | **Reference** |
| --- | --- | --- |
| pCAB18 | IPTG-inducible low copy vector with Tac promotor, Amp^R^ | (4) |
| pRH800 | IPTG-inducible medium copy vector with Tac promotor, Amp^R^ | (5) |
| pKT25 | low copy vector for cloning fusion proteins to the N-terminal T25 fragment of CyaA; lac-promotor; Kan^R^ | (6) |
| pKNT25 | low copy vector for cloning fusion proteins to the C-terminal T25 fragment of CyaA; lac-promotor; Kan^R^ | (6) |
| pUT18 | high copy vector for cloning fusion proteins to the  N-terminal T18 fragment of CyaA; lac-promotor; Amp^R^ | (6) |
| pUT18C | high copy vector for cloning fusion proteins to the  C-terminal T18 fragment of CyaA; lac-promotor; Amp^R^ | (6) |
| pKT25-zip | pKT25 with leucin zipper insert; control plasmid | (6) |
| pUT18C-zip | pUT18C with leucin zipper insert; control plasmid | (6) |
| pETDuet-1 | Overexpression plasmid with 2 MCS under T7 promotor control; Amp^R^ | Merck |
| pKD13 | Template for the amplification of the FRT flanked-kan cassette, *pir*-dependent *oriR*γ, Amp^R^, Kan^R^ | (7) |
| pKD3 | Template for the amplification of the FRT flanked *cm* cassette, *pir*-dependent oriRγ, Amp^R^, Cm^R^ | (7) |
| pKD4 | Template for the amplification of the FRT flanked-kan cassette, *pir*-dependent oriRγ, Amp^R^, Kan^R^ | (7) |
| pKD46 | Helper plasmid encoding λ red recombinase system; arabinose-inducable promotor, temperature-sensitive *ori*, Amp^R^, | (7) |
| pSUB11 | Template for the amplification of the FRT flanked-3xFLAG::kan cassette, *pir*-dependent oriRγ, Amp^R^, Kan^R^ | (8) |
| pCP20 | Helper plasmid encoding FLP recombinase  heat-inducible promotor, temperature-sensitive *ori,* Amp^R^, Cm^R^ | (7) |
| pAP118 | pCAB18::*rdcA^K82A^-rdcB* | (1) |
| pRON1 | pUT18::*rdcA^NTD^* | (1) |
| pRON2 | pUT18C::*rdcA^NTD^* | (1) |
| pRON3 | pKT25::*rdcA^NTD^* | (1) |
| pRON4 | pKNT25::*rdcA^NTD^* | (1) |
| pRON5 | pUT18::*rdcA^CTD^* | (1) |
| pRON6 | pUT18C::*rdcA^CTD^* | this study |
| pRON7 | pKT25::*rdcA^CTD^* | this study |
| pRON8 | pKNT25::*rdcA^CTD^* | this study |
| pRON10 | pETDuet-1::*rdcA-his_6_* (MCS1) | this study |
| pRON11 | pETDuet-1::*rdcA^K82A-^his_6_* (MCS1) | this study |
| pRON12 | pETDuet-1::*rdcA^T103D-^his_6_* (MCS1) | this study |
| pRON20 | pCAB18::*rdcA* | this study |
| pRON25 | pCAB18::*rdcA^K82A^* | this study |
| pRON26 | pUT18::*rdcA^pos^* (E8R, E12R, D725R, D731R) | this study |
| pRON27 | pKT25::*rdcB^neg^* (R218E, R271E, R277E) | this study |
| pRON28 | pCAB18::*rdcA^pos^-rdcB*  (RdcA: E8R, E12R, D725R, D731R) | this study |
| pRON29 | pCAB18::*rdcA-rdcB^neg^*  (RdcB: R218E, R271E, R277E) | this study |
| pRON30 | pCAB18::*rdcA^pos^-rdcB^neg^*  (RdcA: E8R, E12R, D725R, D731R; RdcB: R218E, R271E, R277E) | this study |
| pVP11 | pKT25::*dgcE* | (1) |
| pVP12 | pKT25::*rdcA* | (1) |
| pVP13 | pKT25::*rdcB* | (1) |
| pVP15 | pUT18::*dgcE* | (1) |
| pVP16 | pUT18::*rdcA* | (1) |
| pVP17 | pUT18::*rdcB* | (1) |
| pVP20 | pUT18C::*dgcE* | (1) |
| pVP21 | pUT18C::*rdcA* | (1) |
| pVP22 | pUT18C::*rdcB* | (1) |
| pVP46 | pKNT25::*dgcE* | (1) |
| pVP49 | pKNT25::*rdcA* | (1) |
| pVP70 | pKNT25::*rdcB* | (1) |
| pVP86 | pCAB18::*rdcA-rdcB* | (1) |
| pVS1826 | pTrc99a::*rdcA-yfp* | (3) |
| pVS1610 | pTrc99a::*rdcA^T103D^-yfp* | (3) |
| pVS1611 | pTrc99a::*rdcA^K82A^-yfp* | (3) |
| pVS1612 | pBAD33::*dgcE-cfp* | (3) |
| pVS1840 | pTrc99a::*yfp-rdcB* | (3) |
| pVS1837 | pBAD-DK-Kan::*cfp-rdcA* | (3) |
| pVS863 | pTrc99a::*dgcE-yfp* | (3) |
| PVS1827 | pTrc99a::*yfp-rdcA* | (3) |
| pXC7 | pTrc99a::*yfp-rdcA-rdcB* | this study |
| pLyW1_A3 | pTrc99a::*mNeonGreen_A3 ycgR_mTurquoise2* | (9) |
| pVS2889 | pTrc99a::*mNeonGreen* | (10) |
| pVS2891 | pTrc99a::*mTurquoise2* | (10) |
| pTrc99a | Amp^R^; pBR ori; pTrc promoter; IPTG inducible | (11) |

Table S4: List of primers used for plasmid cloning. Primers with a phosphorylation at the 5’ end (indicated as P) were used to mutate existing plasmids with a backbone PCR.

| **Insert (vector)** | **Sequence (5’-3’)** |
| --- | --- |
| *rdcA^NTD^*  (pUT18, pUT18C, pKT25, pKNT25) | GCCTCTAGAGTACACACAGACCCTGTATGAG |
|  | GCTGGATCCCGGTTACGATCCTGTTGATCGAA |
| *rdcA^CTD^*  (pUT18, pUT18C, pKT25, pKNT25) | GCCTCTAGAGAGTGACGACGCCGACCAGGTG |
|  | GCCGGTACCCGATATCGTTCTGCCGTGAAAAG |
| *rdcA*::his_6_*, rdcA^K82A^*::his_6_*, rdcA^T103D^*::his_6_ (pETDuet-1 MCS1) | GCCCATGGGATACACACAGACCCTGTATGAG |
|  | GCGGAATTCTCAGTGGTGATGATGGTGATGATATCGTTCTGCCGTGAAAAG |
| *rcdA, rdcA^NTD^, rdcA^CTD^,*  *rdcA-rdcB* (pCAB18) | CGCGAATTCATGTACACACAGACCCTGTATG |
|  | CGCGAATTCATGAGTGACGACGCCGACCAGGTG |
|  | GACAAGCTTTTAGTTACGATCCTGTTGATCGAA |
|  | GACAAGCTTTTAATATCGTTCTGCCGTGAAAAG |
|  | GACAAGCTTTTACGCCAAATTTACAGAGAAG |
| *rdcA^pos^*  E8R, E12R, D725R, D731R  (pUT18, pCAB18) | **P**- TTTCTCGCCAACAGTTGCAGTTACTGGAAAAAATGCCTCTCTCTGTACCC |
|  | **P**- GCTGTAACAGGCGTTCAGCACGCTGACTTAAACGATACAGGGTCTGTGTG |
|  | **P**- GGATTTACACCCGTGCCCAGCTGTTACGCGATCGTATTCAAACACTTTTC |
|  | **P**- AGCCGTGAGTCATCACAGTCTGCTGCAATTGCTGCTGCAT |
| *rdcB^neg^*  R218E, R271E, R277E  (pKT25, pCAB18) | **P**- CGCTGGGGATGCCAGAACTCTGTTCAGCGCAGGAACTGGCGGGGGCAATG |
|  | **P**- CGACAGTTTGCCAGCGTTGGGTCACTGACCAGCAAACGGGTTG |
|  | **P**- CGGCACCGACCACGGAACACTATTACCATCAG |
|  | **P**- CATTGATACTCTCTGCCAGATGTTGTGTCGC |
| *yfp-rdcA-rdcB* (pTrc99a) | TCACACAGGAAACAGACCATGTACTAGTGAAGGAGTGTGCCATGGGTATGGTGAGCAAGGGC (Amplify *yfp* with overhangs to pTrc99a) |
|  | ATGGACGAGCTGTACAAGGGAGGTGGAGGATCCGTGTACACACAGACCCT (Amplify *rdcA* with GGGGS linker and GTG start codon for N-terminal *yfp* fusion) |
|  | TCCGCCAAAACAGCCAAGCTTTCAATATCGTTCTGCCGTG (Amplify yfp without stop codon; includes overhangs complementary to *rdcA*) |
|  | CCGCCAAAACAGCCAAGCTTTTACGCCAAATTTACAGAGAAG (Amplify *rdcB* with overhangs to pTrc99a) |

**References**

1.	Pfiffer V, Sarenko O, Possling A, Hengge R. 2019. Genetic dissection of *Escherichia coli*’s master diguanylate cyclase DgcE: Role of the N-terminal MASE1 domain and direct signal input from a GTPase partner system. PLoS Genet 15:e1008059.

2.	Serra DO, Richter AM, Hengge R. 2013. Cellulose as an architectural element in spatially structured *Escherichia coli* biofilms. J Bacteriol 195:5540–5554.

3.	Suchanek VM. 2017. Role of motility and its regulation in *Escherichia coli* biofilm formation. PhD Thesis.

4.	Barembruch C, Hengge R. 2007. Cellular levels and activity of the flagellar sigma factor FliA of *Escherichia coli* are controlled by FlgM-modulated proteolysis. Mol Microbiol 65:76–89.

5.	Lange R, Hengge-Aronis R. 1994. The cellular concentration of the sigma S subunit of RNA polymerase in *Escherichia coli* is controlled at the levels of transcription, translation, and protein stability. Genes Dev 8:1600–1612.

6.	Karimova G, Pidoux J, Ullmann A, Ladant D. 1998. A bacterial two-hybrid system based on a reconstituted signal transduction pathway. Proc Natl Acad Sci USA 95:5752–5756.

7.	Datsenko KA, Wanner BL. 2000. One-step inactivation of chromosomal genes in *Escherichia coli* K-12 using PCR products. Proc Natl Acad Sci USA 97:6640–6645.

8.	Uzzau S, Figueroa-Bossi N, Rubino S, Bossi L. 2001. Epitope tagging of chromosomal genes in *Salmonella*. Proc Natl Acad Sci USA 98:15264–15269.

9.	Wang L, Malengo G, Sanches-Medeiros A, Chen X, Pietsch J, Teteneva N, González Sierra S, Hammond MC, Sourjik V. 2026. Toolbox of FRET-based c-di-GMP biosensors and its FRET-To-Sort application for genome-wide mapping of c-di-GMP regulation. Nat Commun 17:2955.

10.	Scarinci G, Ariens J-L, Angelidou G, Schmidt S, Glatter T, Paczia N, Sourjik V. 2024. Enhanced metabolic entanglement emerges during the evolution of an interkingdom microbial community. Nat Commun 15:7238.

11.	Amann E, Ochs B, Abel K-J. 1988. Tightly regulated tac promoter vectors useful for the expression of unfused and fused proteins in *Escherichia coli*. Gene 69:301–315.
